# 3D structural modeling using seismic data and well logs for Khatatba reservoir in Matruh-Shushan Basin, North Western Desert, Egypt

**DOI:** 10.1038/s41598-023-47487-w

**Published:** 2023-11-17

**Authors:** Amr M. Eid, Walid M. Mabrouk, Mohammed Amer, Ahmed Metwally

**Affiliations:** Geophysics Department, Faculty of Science, Cairo University, Giza, 12613 Egypt

**Keywords:** Solid Earth sciences, Geophysics

## Abstract

Middle Jurassic reservoirs present challenges in the northern segment of the Western Desert due to geometric uncertainties arising from structural configurations, lateral facies variations, diverse lithologies, and heterogeneous reservoir quality. Consequently, this study employed an intricate approach, constructing detailed 3D geostatic models by amalgamating diverse datasets, including 2D seismic sections and digital well-logs. The focus of these 3D models was on the Khatatba Formation (Upper-Safa Member, Kabrit Member, and Lower-Safa Member) in Matruh—Shushan Basin in the North Western Desert. The objectives encompassed assessing hydrocarbon potential, precisely estimating reserves, formulating development and exploration strategies, and identifying prospective drilling locations. The resultant structural model revealed a compartmentalized region marked by major and minor NE–SW trending normal faults, establishing structurally advantageous locations for hydrocarbon trapping within the study area. Petrophysical analyses highlighted the promising potential of the Upper-Safa Member as a reservoir, featuring porosity values ranging from 10 to 18%, peaking in the northeast sector, volume of shale (Vsh) between 15 and 24%, water saturation (Sw) spanning from 18 to 53%, and increased sand thickness towards the eastern section. Similarly, the Lower-Safa Member demonstrated favorable reservoir attributes, including porosity values ranging from 10 to 16%, with higher values in the southeastern part, Vsh between 17 and 28%, and Sw varying from 15 to 47%. The study findings underscored the hydrocarbon potential in the northeast block of the study area for the Middle Jurassic Khatatba Formation. These insights contribute valuable information for decision-making in exploration and production endeavors within the basin.

## Introduction

As hydrocarbon exploration ventures into intricate geological environments, the need for systematic, coordinated approaches grows. Seismic interpretation is key, aiding in understanding subsurface structures, formations, and identifying potential hydrocarbon reservoirs and traps^1^. Simultaneously, petrophysical analysis characterizes reservoir rock properties like porosity, permeability, and fluid saturation, essential for assessing hydrocarbon storage and flow potential. In complex geological settings, the synergy of seismic interpretation and petrophysical analysis is vital, facilitating more accurate and efficient hydrocarbon exploration and production to meet global energy demands^2^.

**Figure 1:**
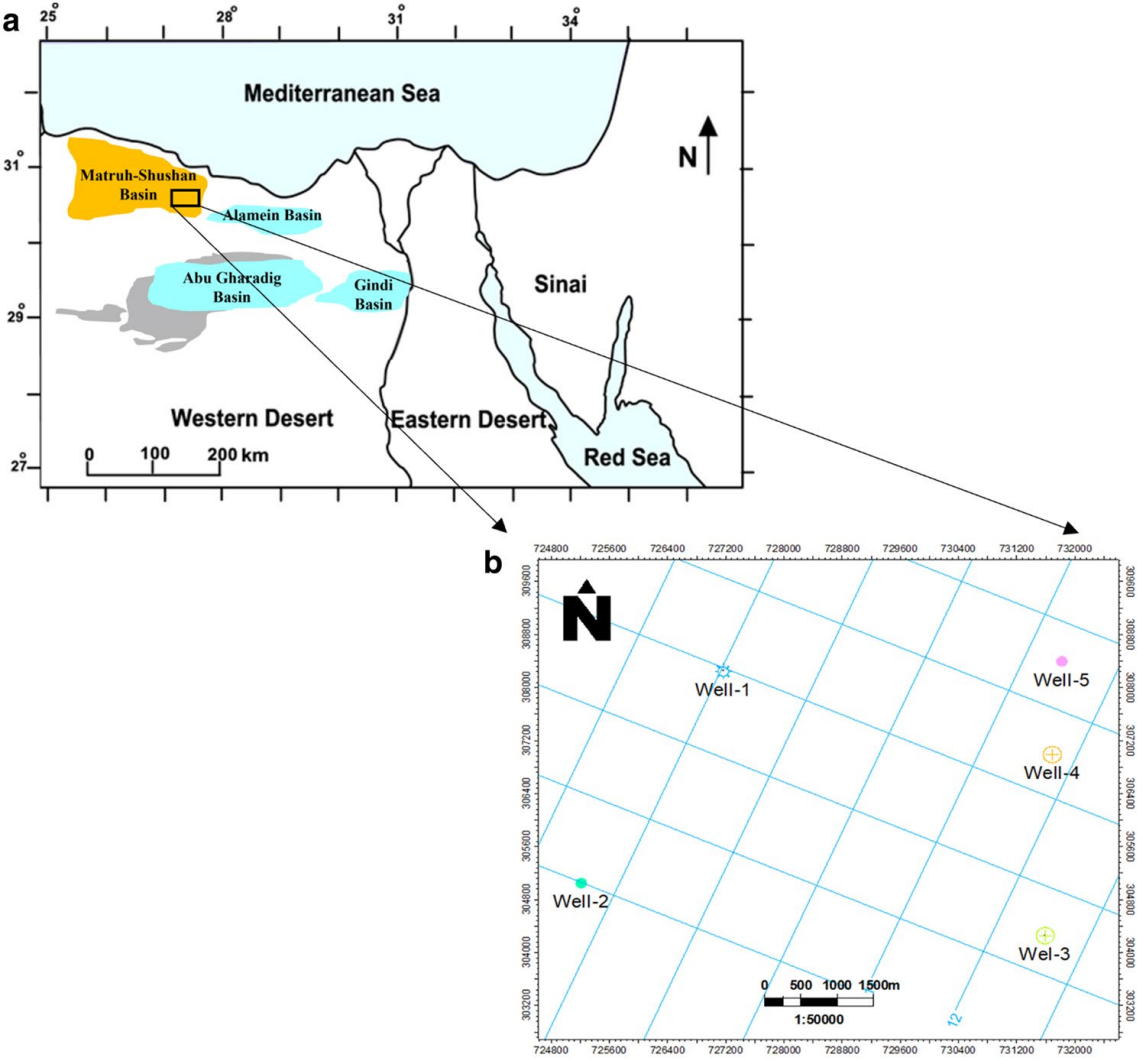
(**a**) Basins distribution in the northern Western Desert (modified after^42^). (**b**) Base map showing the available seismic lines and the well locations.

The primary objective of this study is to construct a 3D structural model and characterize the reservoirs within the Jurassic Upper and Lower Safa Members (Khatatba Formation) of the Matruh-Shushan Basin, situated in Egypt's North Western Desert shown in Fig. 1a. Three-dimensional geological modeling is essential for representing geological features such as the configurations of geological structures, the interconnections between geological entities, and the spatial patterns of geophysical and geochemical properties within geological formations using a suitable computer data format. Our approach involves the interpretation of 2D seismic data and well-log information to achieve this goal. Advanced software (Petrel-2017) aids in the interpretation of seismic data, enabling the mapping of geological formations and a deeper understanding of the basin's structural framework, ultimately assisting in pinpointing promising drilling locations. Petrophysical analysis plays an integral role in assessing the hydrocarbon potential of the basin. This analysis involves the examination of well logs and core samples to determine essential reservoir properties, including porosity, permeability, and fluid saturation. These evaluations provide critical insights into the quality and productivity of reservoir rocks, as well as estimates of hydrocarbon volumes within the Middle Jurassic Khatatba Formation.

Additionally, this study aims to provide insights into the methodologies and workflows employed in the interpretation of seismic data and the analysis of petrophysical properties. It's important to note that assessing the hydrocarbon potential of the Matruh-Shushan Basin is a complex task due to the basin’s geological complexity, characterized by a range of depositional environments and tectonic influences. This complexity presents both challenges and opportunities for accurately estimating the basin's hydrocarbon potential. The study focuses on thirteen seismic lines (Inlines and cross-lines) and five wells (well-1, well-2, well-3, well-4, and well-5) within the Matruh-Shushan Basin, with their locations depicted in Fig. 1b.

The research area is situated within the expansive North Western Desert of Egypt, specifically within the notable Matruh-Shushan Basin, a significant geological and geographical aspect of the region. It has gained recognition for its substantial hydrocarbon potential, making it a focal point of interest for the oil and gas industry^3,4^. The basin's location in the North Western Desert significantly influences its geological attributes, encompassing sedimentary rock formations, diverse structural elements, and the presence of valuable hydrocarbon reservoirs and source rocks^5^. This unique setting presents both challenges and opportunities, rendering the Matruh-Shushan Basin an intriguing and promising area for geological exploration, resource evaluation, and hydrocarbon production activities.

## Geological setting

The Western Desert extends west of the Nile River and covers more than 700,000 km^2^, accounting for approximately two-thirds of Egypt’s total land area. In the southernmost region of the Western Desert, the pre-Paleozoic basement is exposed, exhibiting a gradual slope towards the north. This slope is accompanied by an increase in the thickness of the sedimentary layers, which consist entirely of formations from the Paleozoic, Mesozoic, and Tertiary to recent times^5^.

More than half of Egypt's daily oil production originates from the Mesozoic-age rift basins located in the northern part of the Western Desert. This includes basins such as Shushan, Matruh, and Alamein.

These basins hold approximately 40% of Egypt's confirmed oil reserves^6^. The Matruh-Shushan Basin is commonly acknowledged for the increased thickness of sedimentary layers in its northern region. This thickening is attributed to a wide basement relief caused by normal block faulting accompanied by minor compressional folding^7^. The Matruh-Shushan Basin experienced extension during the Jurassic and Early Cretaceous periods, which was subsequently followed by inversion during the Late Cretaceous and early Tertiary periods^8^. The geological history and the influencing tectonic events led to E-W and ENE-WSW trending faults during the Cretaceous, and Jurassic in the Northern Egyptian Western Desert^1,9^.

The sedimentary section of the northern Western Desert encompasses a wide range of ages, spanning from the Early Paleozoic era to the Recent period^10^. Within this section, four significant sedimentary cycles have been identified: the Carboniferous, Late Jurassic, Middle and Late Cretaceous, and Middle Miocene^11^. These cycles were characterized by distinct southward transgressions, indicating a maximum advance of the sea. Conversely, regressive episodes were predominant during the Triassic and Early Jurassic, with northward retreats. These regressive episodes continued into the Early Cretaceous and Late Eocene periods^12^.

The sedimentary sequence in the Western Desert consists of a succession of depositional cycles that are influenced by both tectonic and eustatic factors as follows^13^:

- The oldest sedimentary rocks exhibit a clastic facies cycle that covers the Paleozoic and Lower Jurassic formations.
- A carbonate section is present in the middle and Upper Jurassic formations.
- The Lower and Upper Cretaceous formations constitute a cycle of clastics, particularly in the Early Cenomanian period.
- In the northern Western Desert, carbonate deposits are observed in the Upper Cenomanian and Upper to Middle Eocene formations.
- The uppermost clastic cycle is represented by the Upper Eocene–Oligocene, Miocene, and newer sections.

**Figure 2:**
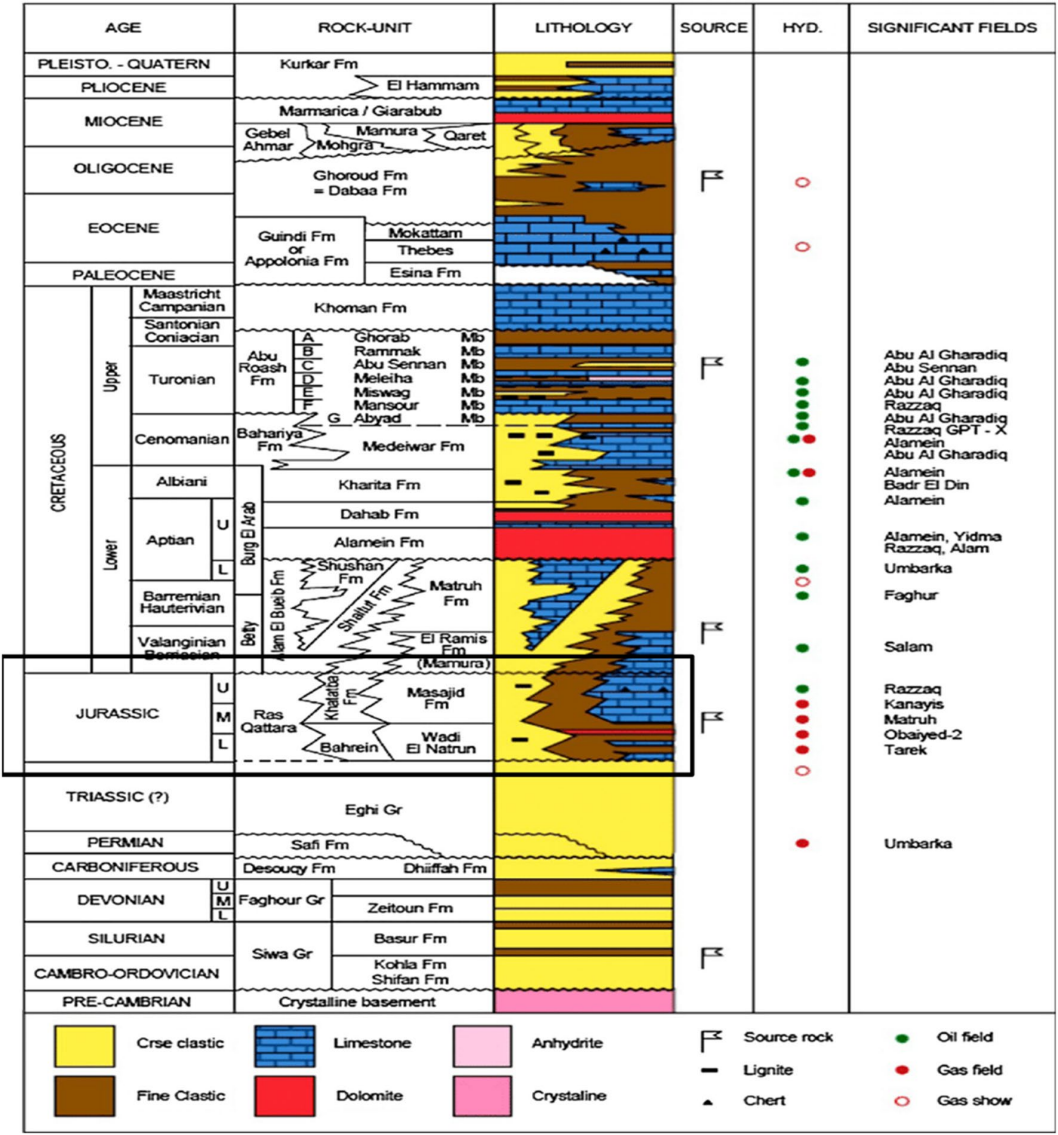
Generalized stratigraphic column of the North Western Desert with highlight to Khatatba Fm including Upper and Lower Safa member. (Modified after^12,13^).

A notable component of the stratigraphic column in the Western Desert is the Khatatba Formation. Generalized stratigraphic column of the North Western Desert with highlight to Khatatba Fm including Upper and Lower Safa member shown in Fig. 2. This formation consists of a thick series of carbonaceous shale interbedded with porous sandstone, coal deposits, and limestone stripes^3^. In the North Western Desert, the Matruh-Shushan Basin contains the Middle Jurassic Khatatba Formation, with a thickness ranging from 283 to 358 m.

The strategic positioning of the northern Western Desert in relation to the source rock makes it an attractive location for petroleum exploration. The organic-rich rocks within the Khatatba Formation are believed to be highly productive as a source of oil and gas, particularly for accumulation within the Khatatba sandstone reservoir rocks^9^.

## Materials and methods

To enhance our understanding of the geological composition within the study region, an in-depth analysis was conducted on thirteen seismic lines and wireline logs from five specific wells. The primary objective was to provide a comprehensive description and characterization of the various structural elements that influence the middle Jurassic Khatatba Formation. Additionally, key horizons such as the Upper Safa, Kabrit, and Lower Safa members were identified and marked along the seismic lines to facilitate the creation of depth structure contour maps. All the wells had log data coverage within the Khatatba Formation which is the main target interval for this study.

**Figure 3:**
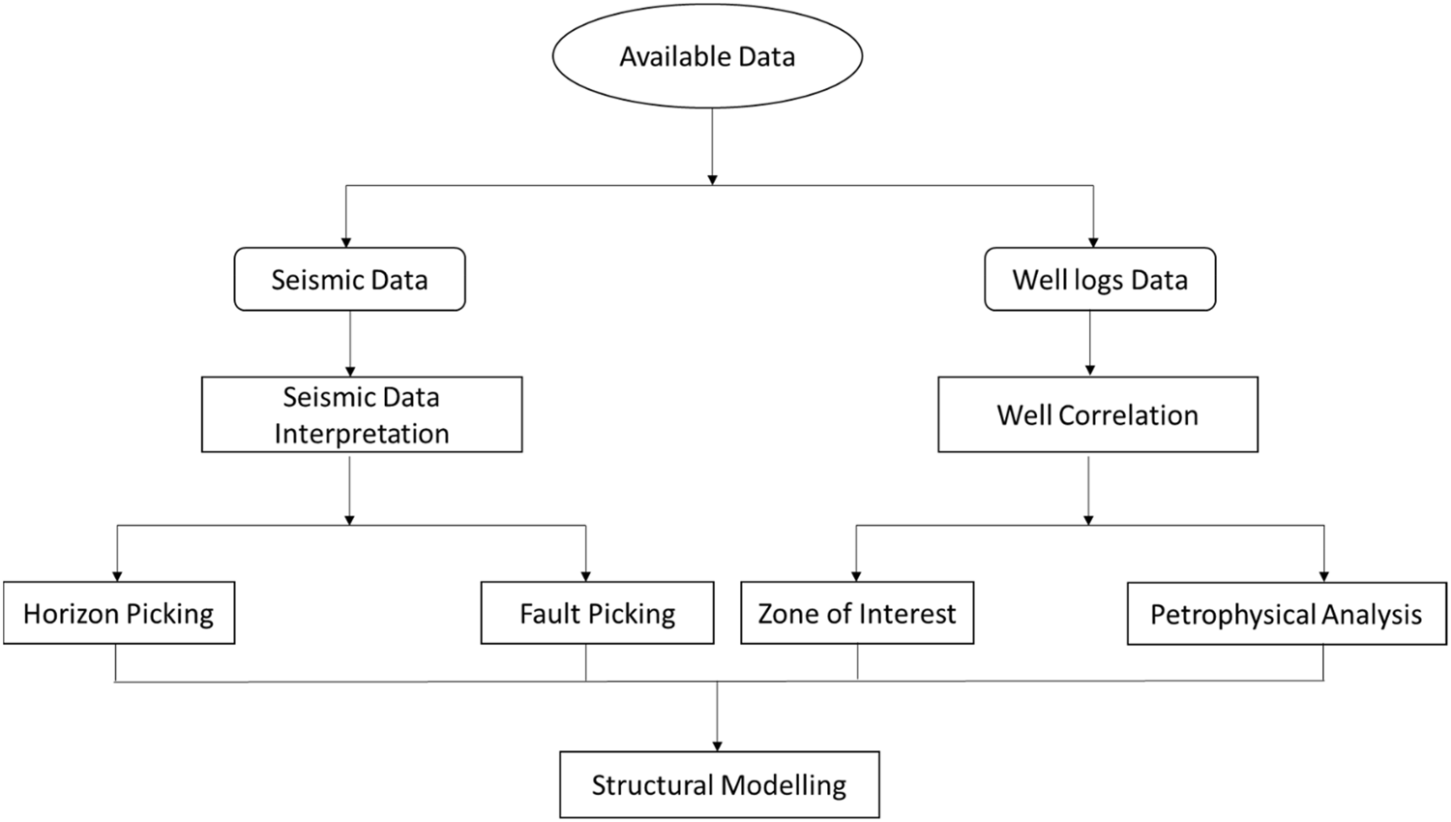
Flowchart of the 3D geological modeling.

The Flowchart of the 3D geological modeling is shown in (Fig. 3). The initial step in identifying horizons typically involves using seismic lines that intersect wells^14^, which allows for a correlation between seismic reflections and the underlying stratigraphy^15^. To achieve local continuity and similarity in character, the horizons were manually picked, facilitating the identification of the specific event of interest in the seismic lines^16^.

Subsequently, the fault heaves are manually identified and marked on their respective positions on the seismic shot point base map, forming a fault pattern. This pattern is used to generate a 3D structural model for the relevant topographical features^17,18^. Well correlation and Petrophysical analysis were done in parallel with the seismic interpretation. The entirety of this study involved the utilization of Petrel™ 2017 Schlumberger to execute each step outlined in the provided flowchart. The data undertook a rigorous quality control (QC) process to verify its accuracy. Subsequently, it was precisely structured within well-defined databases, ensuring consistency and compatibility across various datasets.

## Results and discussion

### Seismic data interpretation

The primary stages in seismic data interpretation involve identifying seismic surfaces and fault detection^19,20^. Within these steps, one can recognize seismic horizons along with structural and stratigraphic characteristics^21,22^. The seismic interpretation procedure consists of various tracks reserved to find an explanation of the seismic sector on the subject of all stratigraphic structures and sequences^19,23,24^. The thirteen seismic sections are used to identify and track three formation tops: Upper Safa Member, Kabrit Member, and Lower Safa Member. Their lateral movement is monitored within the seismic data. In all of the seismic sections, faults are interpreted and traced. The seismic sections are oriented in northeast–southwest directions (Fig. 1).

**Figure 4:**
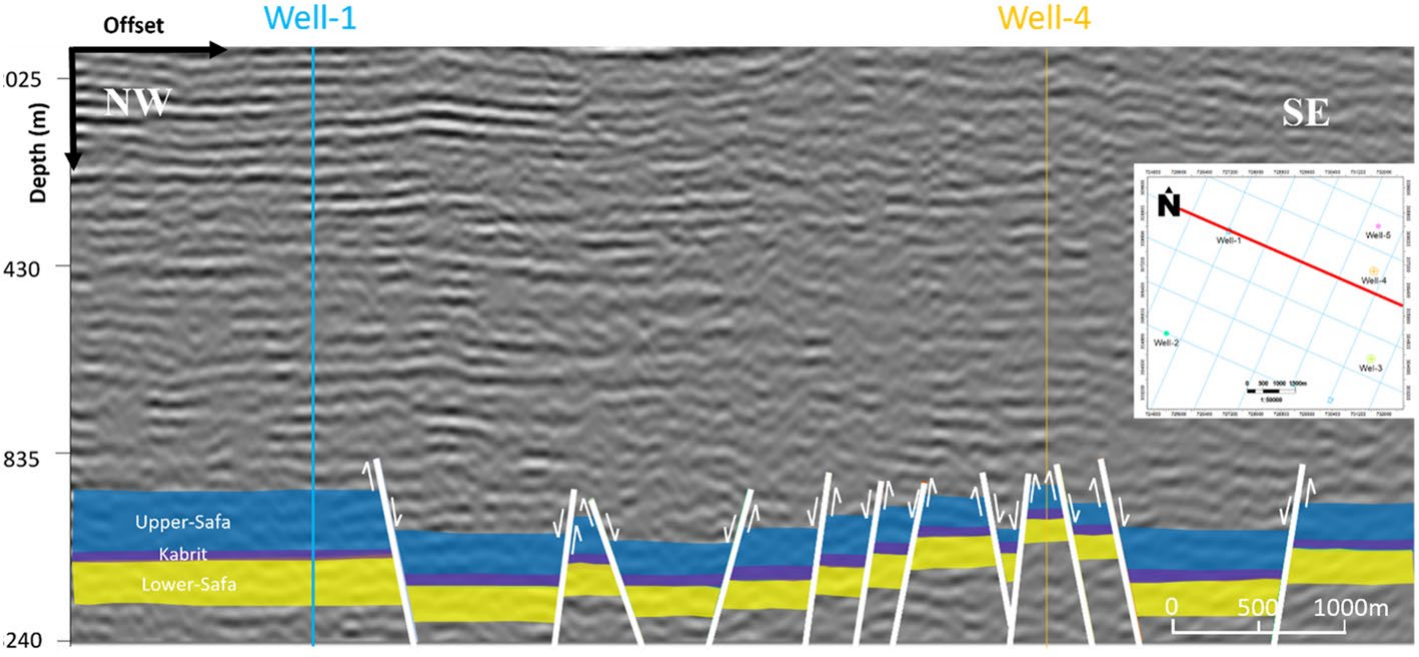
Interpreted seismic section (Line-4).

**Figure 5:**
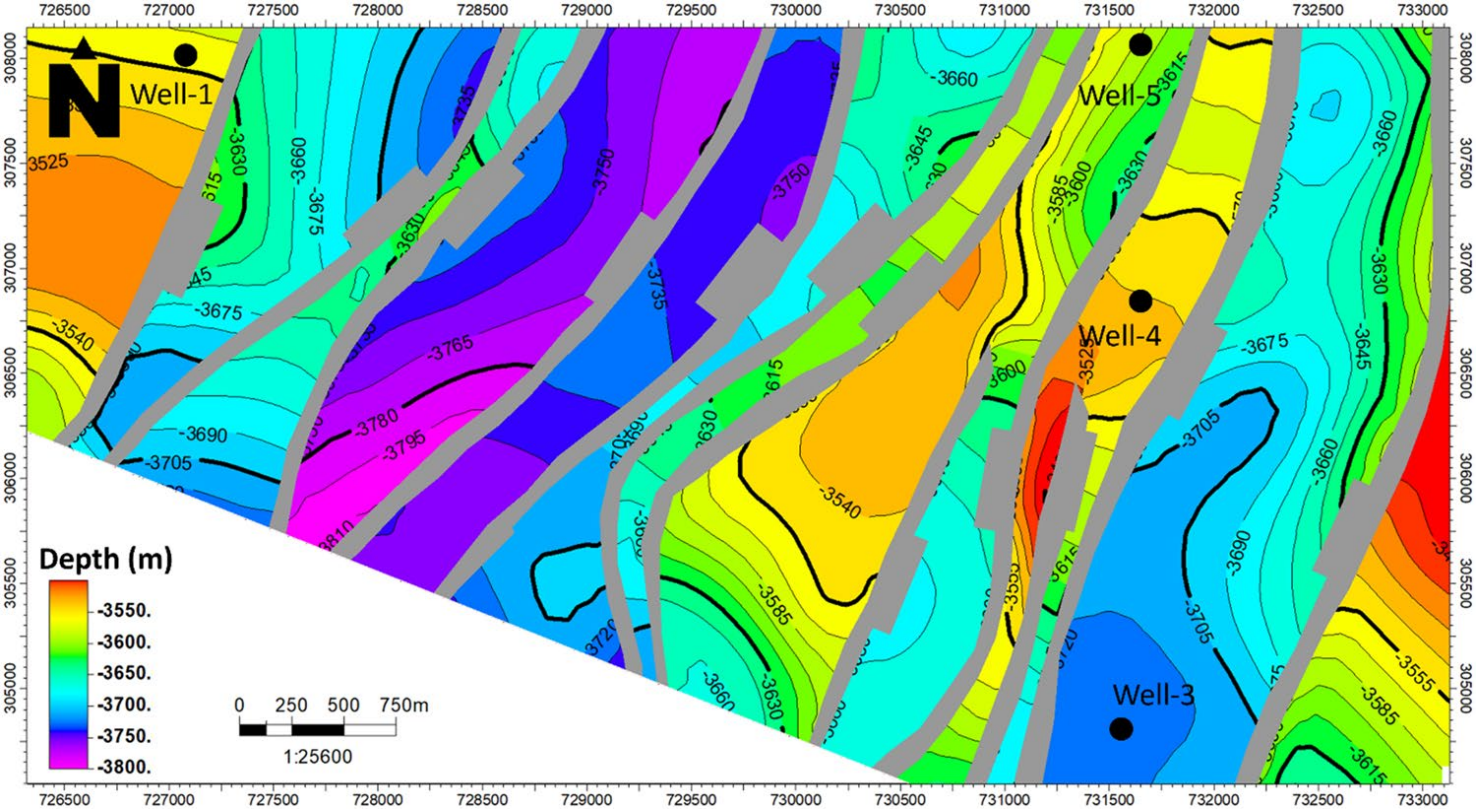
Depth structure contour map of Upper-Safa Fm.

**Figure 6:**
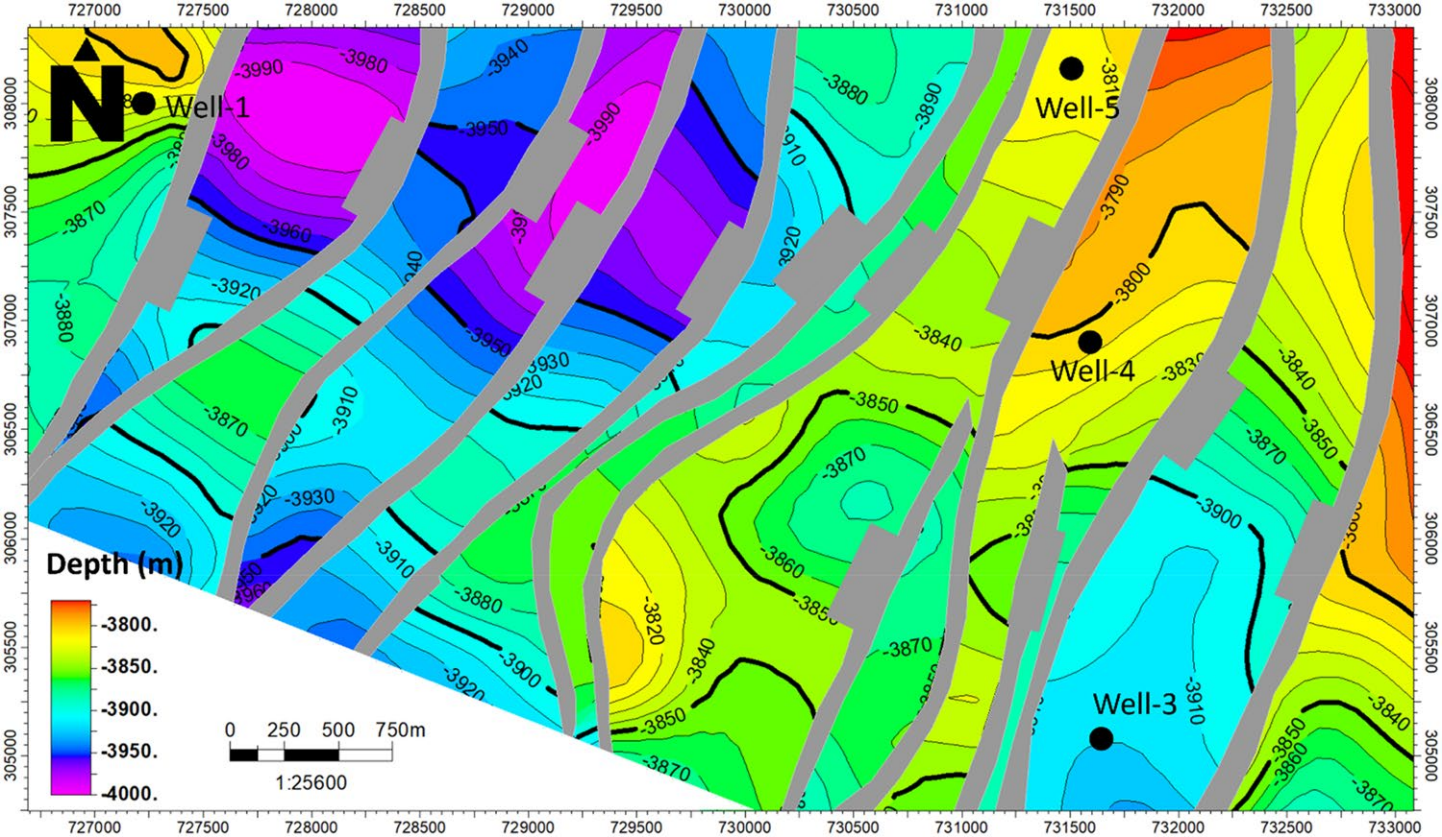
Depth structure contour map of Lower-Safa Fm.

The study area is located within an extensional regime, resulting in a predominant normal-related structure known as a group of step faults. In this extensional regime, normal faults develop and evolve. The seismic section reveals the presence of twelve faults, adding complexity to the research area. These faults are identified based on abrupt changes in reflector conditions and disruptions or the absence of reflections below the faults. Petrel software was used for the seismic interpretation. The interpretation of the seismic sections shows the trend of the faults in the northeast–southwest direction. All three formations appear to be affected by a series of normal faults that correspond with the geologic setting of the area. The interpreted normal faults and the structural closures of the three Jurassic formations were observed as shown in (Fig. 4). Depth structure contour maps for the Upper Safa Member and Lower Safa Member were created (Figs. 5, 6). These maps reveal a pattern of normal block faulting that trends in a northeast-southwest direction and this agreed with^25^ which stated that the primary trapping mechanism resulted from structural closure, arising from faulting and folding processes that occurred during the Late Cretaceous to the post-Middle Eocene inversion events.

Within the depicted depth maps of the Upper and Lower Safa Formations (illustrated in Figs. 5 and 6), a total of twelve normal faults are distinctly delineated across the expanse, influencing both formations and giving rise to a distinctive step-like configuration. This structural pattern is recognized for its potential in hydrocarbon production from the elevated areas, known as horsts, provided that the faulting is effectively sealed. Encouragingly, the likelihood of these faults being sealed is heightened by the presence of shale interlayers within the formations, acting as a natural barrier. To ascertain the precise hydrocarbon reserves present, the petrophysical analysis assumes a pivotal role by leveraging information derived from the well logs.

### Well correlation

Well log data correlation plays a pivotal role in achieving a high-quality 3D geological model within the Petrel software. This process involves organizing and categorizing well log data for simple 2D visualization. It primarily focuses on facilitating comparisons between wells, resulting in the creation of well sections and a comprehensive set of correlated wells.

**Figure 7:**
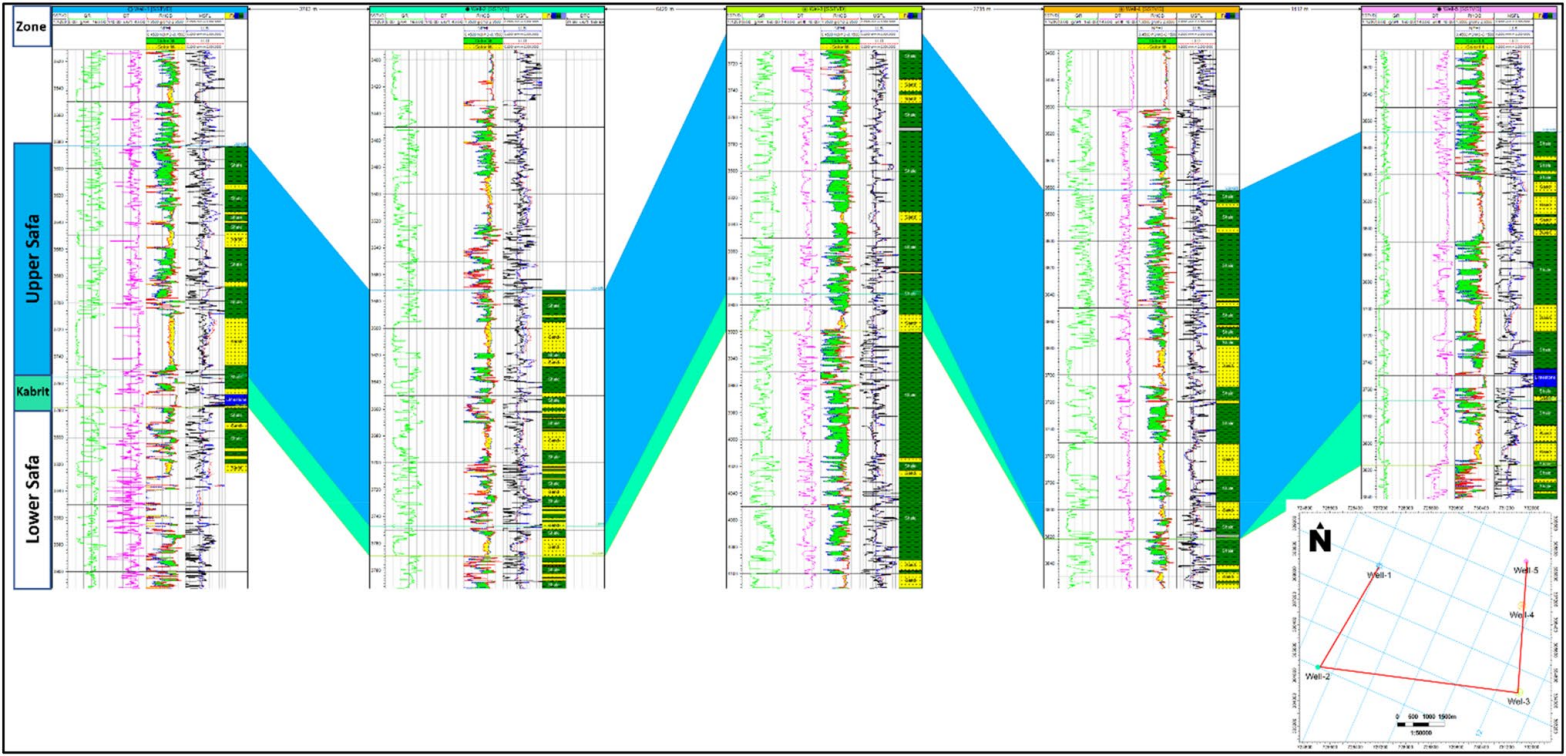
Well correlation chart depicting the geological relationships and stratigraphic alignments within the study area.

**Figure 8:**
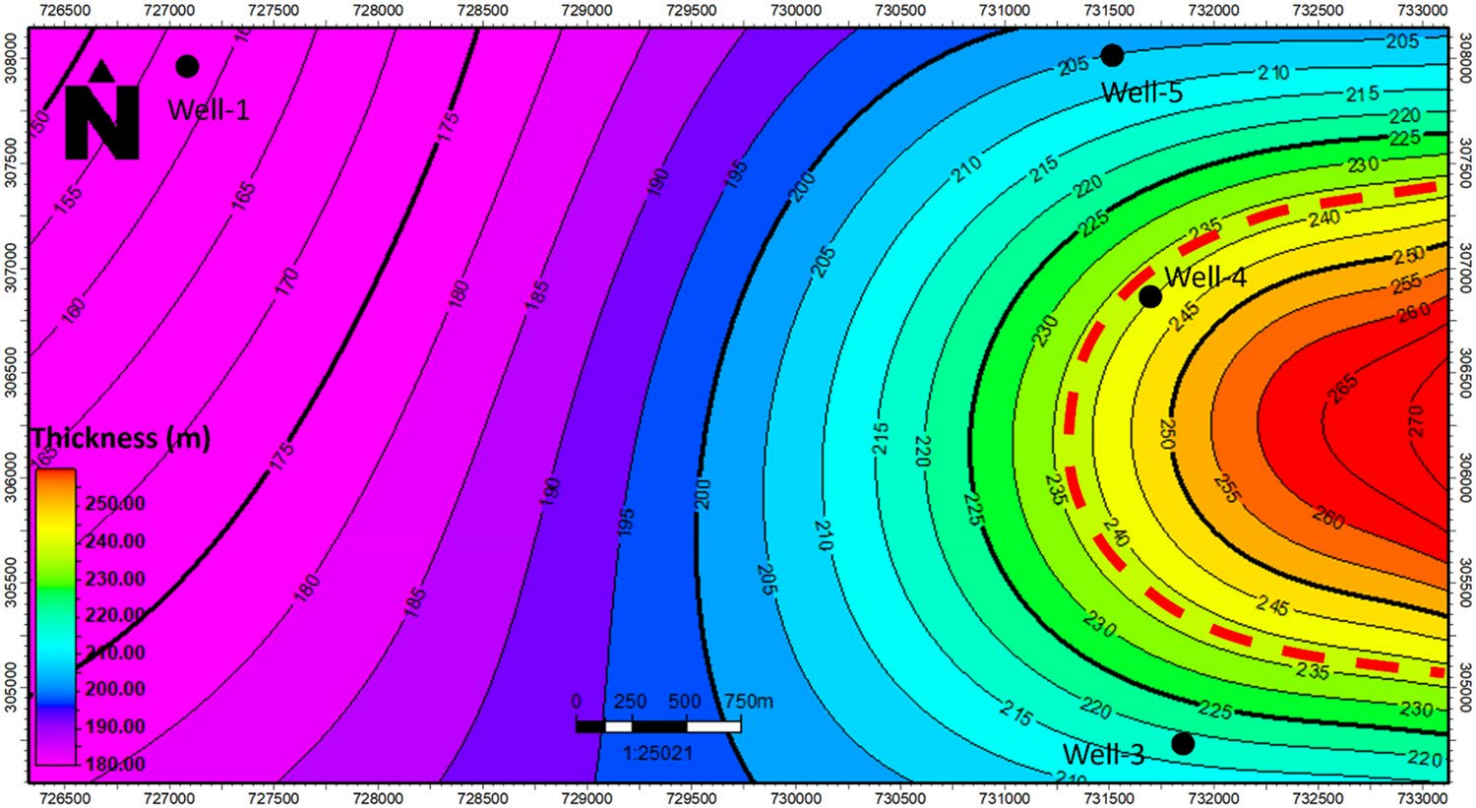
Sandisolith map illustrating the Upper-Safa Formation, with a highlighted area showing eastward thickening, indicated in red.

In this study, a well correlation was conducted to illustrate variations in both thickness and reservoir properties across different geological units within the Jurassic reservoir^26,27^. The findings are presented in Fig. 7. Figure 7 showcases a vertical section encompassing wells well-1, well-2, well-3, well-4 and well-5, elucidating the thickness of Jurassic units and alterations in petrophysical properties within the reservoir units. Observing Fig. 7, it becomes evident that each well is equipped with gamma-ray (GR), porosity (NPHI), density (RHOB), sonic time (DT), and resistivity data. The uppermost Upper-Safa formation has been established as the reference top for each Jurassic unit. Through the establishment of correlations among the wells, it becomes evident that the Upper-Safa zone exhibits favorable petrophysical properties. This observation aligns with the description that the Upper Safa Member, a component of the Middle Jurassic Khatatba Formation in the Shushan Basin within the Obaiyed Field, is primarily composed of shale, with notable sandstone reservoir intervals known as unit A and unit B, both of which serve as productive gas pay zones^15,28^. Consequently, this zone garners attention as a significant area of interest within the Jurassic reservoir. Furthermore, the study reveals that this zone exhibits greater thickness increments toward the east trend, contrasting with the units in the west as observed along the vertical section. Figure 8. Shows the Sandisolith map of the Upper-Safa formation.

### Petrophysical analysis

The primary objective of conducting a petrophysical analysis is to extract valuable information and data from the well logs^29^. Petrophysical analysis of well logs plays a crucial role in characterizing reservoir rocks and is considered highly valuable. The productivity of wells in hydrocarbon-bearing reservoirs relies on various petrophysical properties such as lithology, porosity, water saturation, permeability, and saturation^30^. The data used in the present research study consists of log curves representing gamma ray, neutron, density, and resistivity logs. An expedited analysis of our data was conducted, followed by the identification of zones that satisfy particular criteria. The foremost determinant is a substantial rise in deep resistivity, whether or not there is a distinct separation between shallow and deep resistivity measurements. This gap indicates erosion of the zone, suggesting a predominantly hydrocarbon composition. Evaluating our selected zone reveals a diminished gamma ray reaction and a merging representation of the φN and ρb logs^1,17,30,31^. The petrophysical parameters are shown in Table 1. These parameters' values were in agreement with 1.

**Table 1:** Petrophysical Evaluation for upper and Lower Safa Fm.

Parameters	Wells/Zones	Well-1	Well-2	Well-3	Well-4	Well-5
Gross thickness	Upper Safa	574.146	574.146	639.764	853.019	656.168
	Lower Safa	927.741	637.45	1267.64	1026.67	1212.97
Net thickness	Upper Safa	222.5	263.3	149.2	306.5	583.9
	Lower Safa	95.25	82	420.8	342.5	986.2
Volume of shale fraction	Upper Safa	0.199	0.234	0.246	0.197	0.16
	Lower Safa	0.25	0.287	0.236	0.276	0.17
Effective porosity fraction	Upper Safa	0.063	0.059	0.067	0.054	0.106
	Lower Safa	0.128	0.116	0.053	0.071	0.169
Water saturation fraction	Upper Safa	0.377	0.185	0.531	0.425	0.196
	Lower Safa	0.157	0.175	0.479	0.252	0.151

The linear gamma-ray index equation was used to determine the shale volume (Eq. 1) and made further adjustments using the Steiber equation (Eq. 2) to obtain accurate shale volume measurements^32,33^. This analysis revealed the presence of both sand and shale intercalations in the Lower and Upper Safa members, which were of particular interest. To determine the effective porosity value relevant information from (Eq. 5) was extracted. Lastly, the water saturation was calculated using the Indonesian model (Eq. 6).

Linear gamma-ray index equation:

1 $${I}_{GR} =\frac{{GR}_{log}- {GR}_{min}}{{GR}_{max} - {GR}_{min}},$$

Steiber equation^34^ to adjust the calculated shale volume:

2 $${V}_{sh} =\frac{0.5 * {I}_{GR}}{1.5 - {I}_{GR}},$$

In which:

$${I}_{GR}=gamma\, ray\, index;\,Vsh=volume \,of \,shale;\,GRLog=gamma \,ray \,reading;\,GRmax=maximum\, gamma\, ray\, (shale);GRmax=minimum \,gamma \,ray\, (clean\, sand\, or \,carbonate).$$

To estimate the effective porosity from logs we will use some equations described in details below in which we will calculate the Total Porosity from ${\varnothing }_{N}$ log and from ${\rho }_{b}$ log which is called ${\varnothing }_{D}$ as follows:

3 $${\varnothing }_{D}=\frac{{\rho }_{m}-{\rho }_{b}}{{\rho }_{m}- {\rho }_{fl}},$$

4 $${\varnothing }_{T}= \frac{{\varnothing }_{N}+ {\varnothing }_{D} }{2} ,$$

5 $${\varnothing }_{eff}= {\varnothing }_{T}-\left( {V}_{sh}* {\varnothing }_{sh}\right),$$

where: $${\varnothing }_{D}=Porosity\, from \,density\, log$$; $${\rho }_{m}=Density \,of \,matrix$$; $${\rho }_{b}= Density \,from \,log$$; $${\rho }_{fl}=Density\, of\, Pore\, fluids$$; $${\varnothing }_{T}=Total\, Porosity;$$ $${\varnothing }_{eff}=Effective \,Porosity;$$ $${V}_{sh}=Volume\, of \,shale$$; $${\varnothing }_{sh}=Porosity \,of \, shale.$$

The Indonesian model was created by^35^ and it is represented by the change in calculated water saturation (${S}_{w}$) as a function of true resistivity (Rt) and reservoir rock shale content.

6 $${S}_{w}={\left[ \frac{{V}_{sh}^{0.5(1-{V}_{sh})}}{{(\frac{{R}_{sh}}{{R}_{t}})}^{0.5}+{(\frac{{R}_{sh}}{{R}_{0}})}^{0.5} } \right]}^{\frac{-2}{n}},$$

### The 3D geological modeling

3D geological modeling involves the creation of a digital representation of the subsurface geology in three dimensions. It integrates diverse geological data, such as well logs, seismic data, and observations, to build an accurate model that depicts the distribution and characteristics of geological features beneath the Earth’s surface^36^. The 3D geological model primarily comprises the area's structural framework, encompassing faults and surfaces, along with the zonation and layering details of the Upper Safa Member, Kabrit Member, and Lower Safa Member. Geostatistical techniques have been employed to model various geological properties, enabling the estimation of values for these properties at locations where no drilling has taken place. Petrel software was utilized to develop a comprehensive 3D geological model by incorporating various parameters, such as shale volume, porosity, facies, fluid saturation, and net to gross thickness.

### Structural modeling

The initial and vital stage in developing a 3D geological model is structural modeling, which involves two parallel approaches. First, it includes creating maps that depict the structural arrangement of formation tops. Second, it involves identifying fault systems that traverse through the reservoir^37^. The construction of a structural model involves a sequential process comprising three key stages: geometry definition, fault framework modeling, and horizon modeling^38^. The initial step involves defining the geometric properties by automatically delineating the X–Y extent of the desired region based on seismic survey data. The subsequent phase entails the creation of fault models derived from interpreted seismic section faults within the depth domain. A pivotal aspect of establishing an effective structural model lies in accurately depicting the interconnectedness of faults, a task that can be fine-tuned through the manipulation of fault sticks^39,40^. The final facet revolves around the modeling of horizons, which is achieved by utilizing interpreted horizons, thickness maps, and well top data specific to the study area^9^.

**Figure 9:**
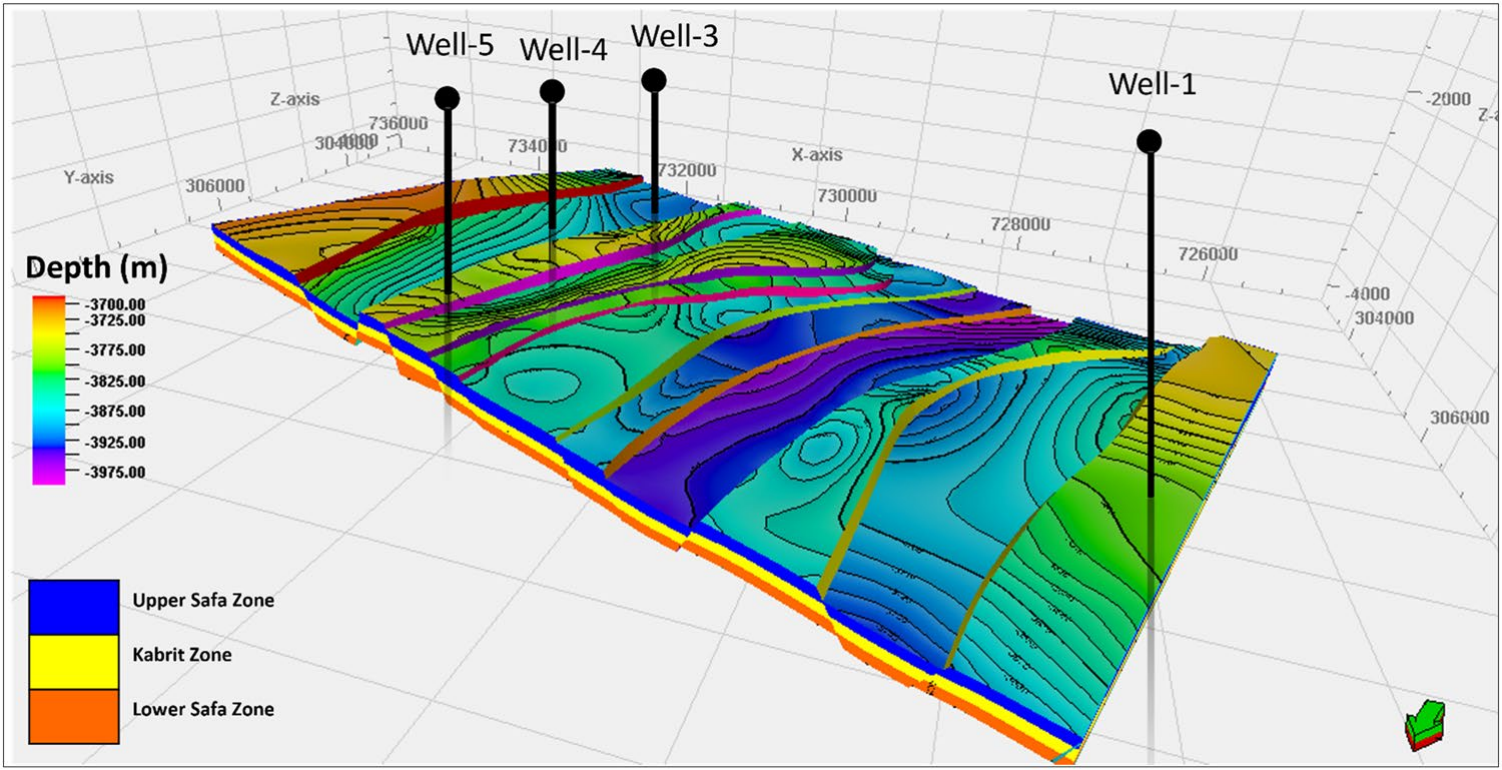
The 3D Structural model of the study area with zones.

The Matruh-Shushan Basin underwent investigation using Petrel software in conjunction with a 2D seismic survey, resulting in the creation of a 3D structural model encompassing the study area, as depicted in Fig. 9. This comprehensive model was specifically developed to encompass three primary formations: Upper-Safa, Kabrit, and Lower-Safa. Within this model, a series of step faults emerged, exerting an influence on all three formations. These step faults exhibited orientations spanning northeast-southwest direction.

**Figure 10:**
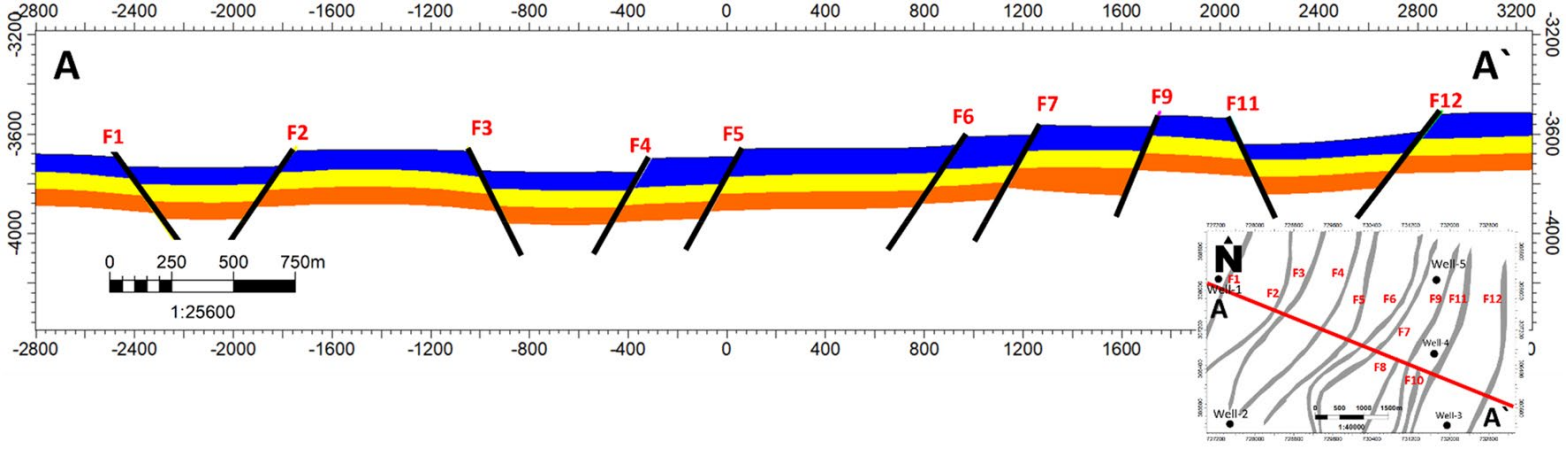
NW–SE integrated cross section A–A′ with zones.

A cross-sectional view marked as NW–SE integrated Cross section A–A′ was meticulously generated, as illustrated in Fig. 10. This cross-section serves to affirm the presence of the aforementioned step faults and effectively delineates the zones influenced by these faulting mechanisms.

### Petroleum system

In the northwestern desert of Egypt, specifically in the Matruh-Shushan Basin, the Upper Safa and Lower Safa formations are considered important reservoir rocks. These formations have gained attention due to their potential as hosts for hydrocarbon accumulations^7^. The Upper Safa Formation is a significant reservoir rock within the Matruh-Shushan Basin. It consists of sandstone units that exhibit favorable porosity and permeability properties. These properties allow the formation to store and transmit hydrocarbons effectively. Similar to the Upper Safa Formation, the Lower Safa Formation also contains sandstone units that can act as reservoir rocks. This formation is located beneath the Upper Safa and exhibits properties conducive to hydrocarbon accumulation. It is considered an important contributor to the hydrocarbon potential of the Matruh-Shushan Basin.

These formations encompass intervals of sandy reservoirs as well as organic-rich shales with the potential to act as source rocks. The Upper Jurassic Masajid Formation, consisting of substantial carbonates, holds promise as potential seal rocks^13^. In the Matruh-Shushan Basin, both shales and limestone units serve as source rocks for oil generation, particularly in the central region of the basin^41^. The faults within this area serve as pathways for migration. The structural traps within this system, developed during the Late Cretaceous to Early Miocene, primarily consist of the inverted hanging walls of tilted blocks and folds, resulting from the inversion of these former half grabens. Additionally, the possibility of stratigraphic traps within the Khatatba Formation should not be discounted, especially given its heterogeneous nature, characterized by fluvial and tidal channel deposits^15^.

The examined region holds a favorable petroleum system with favorable physical characteristics, thus advocating for the consideration of further development well drilling. Based on the investigation, additional prospective zones that present suitable locales for the accumulation of hydrocarbons may be found towards the northeast trend of the study area.

## Summary and recommendations

The seismic interpretation was conducted utilizing Petrel™ 2017 Schlumberger. The seismic analysis highlights the existence of twelve faults, introducing intricacies to the study zone. Significantly, all three formations within the area display the influence of a sequence of normal faults, aligning coherently with the region's geological framework. In the examined area, a notable arrangement of twelve distinct normal faults shapes the landscape, influencing both formations and creating a characteristic step-like structure. This unique geological setup holds promise for potential hydrocarbon production from elevated zones, termed horsts, given effective fault sealing. Encouragingly, the possibility of such sealing is augmented by the existence of shale interlayers in the formations, serving as inherent barriers.

To accurately determine the extent of hydrocarbon reserves, the petrophysical analysis was conducted on well log data. This study involved well correlation to elucidate changes in thickness and reservoir characteristics across distinct geological units within the Jurassic reservoir. The analysis highlighted the Upper-Safa zone as particularly notable for its favorable petrophysical attributes.

Moreover, the investigation unveiled a trend where this zone demonstrated increasing thickness towards the east, contrasting with the units in the west as indicated by the vertical section. This underscores the significance of the Upper-Safa zone and provides insights into its potential for hydrocarbon exploration and exploitation. Petrophysical Evaluation for upper and Lower Safa formations revealed that Upper Safa has a volume of shale of about 0.19, effective porosity of about 0.11, and hydrocarbon saturation ranges from 0.6 to 0.72. while Lower Safa has a volume of shale of about 0.21, effective porosity about 0.12, and hydrocarbon saturation ranges from 0.58 to 0.68.

The Matruh-Shushan Basin was analyzed through a combination of Petrel™ 2017 Schlumberger and a 2D seismic survey. This comprehensive approach led to the development of a 3D structural model that covers the entire study area. In creating this comprehensive model, a focused effort was made to encompass three key formations: Upper-Safa, Kabrit, and Lower-Safa. The model's depiction revealed a series of step faults that significantly impacted all three formations, characterized by orientations spanning the northeast-southwest direction. A meticulously crafted cross-sectional view, labeled as the NW–SE integrated Cross section A–A′, has been generated for confirmation. This cross-section serves the purpose of confirming the existence of the previously mentioned step faults and precisely outlining the areas affected by these faulting processes.

In light of the promising findings from our study, we recommend the strategic drilling of new development wells aimed toward the northeast direction within the study area. This direction aligns with the trend of favorable geological features identified in our analysis. By expanding exploration efforts in this direction, we can potentially tap into untapped hydrocarbon reserves and further enhance our understanding of the subsurface dynamics. This proactive approach aligns with our goal of maximizing the reservoir's potential and optimizing hydrocarbon recovery.

## Conclusion

The main points of our journey are summarized in this final section, together with their possible implications for the fluid environment of hydrocarbon exploration in the Matruh-Shushan Basin. The following describes these key revelations:

- The study provides valuable insights into the hydrocarbon potential and structural configuration of the Matruh-Shushan Basin. The main structure of the basin was found by interpreting thirteen seismic lines. It is mostly made up of a network of step faults that affect the Upper-Safa, Kabrit, and Lower-Safa formations.
- Subsequent well correlation and petrophysical analysis of five wells indicated that the Upper-Safa formation exhibits the most promising reservoir quality.
- A comprehensive 3D model of the study area was meticulously constructed to visualize how these faults impact the three formations and act as effective hydrocarbon traps throughout various locations in the basin.
- Adding seismic interpretation and petrophysical analysis to the 3D model revealed details that were hidden below the surface. This helped us learn more about the unique Jurassic reservoirs in the study area.
- The 3D model also provided spatial insights into the arrangement of these subsurface horizons, aiding in identifying potential reservoirs for future drilling operations.
- The Upper-Safa and Lower-Safa reservoirs emerged as highly prospective prospects due to their impressive porosity levels and minimal water saturation levels.

The study results and findings can be considered as an analog and could be generalized on nearby basins in the Northern Western Desert.

## Data Availability

The data that support the findings of this study are available from (The Egyptian General Petroleum Cooperation) but restrictions apply to the availability of these data, which were used under license for the current study, and so are not publicly available. This data is available from the corresponding author upon reasonable request and with permission of (The Egyptian General Petroleum Cooperation).
